# Impact of multi-strain probiotics supplementation on growth, immune responses and physiological traits in backyard poultry of Andaman and Nicobar Islands, India

**DOI:** 10.3389/fmicb.2025.1625167

**Published:** 2025-08-12

**Authors:** Neha Halder, Siddhartha Narayan Joardar, Jai Sunder, Arun Kumar De, Debasis Bhattacharya, Ahmed Abd El Wahed, Rea Maja Kobialka, Madathiparambil Gopalakrishnan Madanan, Samiran Mondal

**Affiliations:** ^1^Division of Animal Science, ICAR-Central Island Agricultural Research Institute (I.C.A.R-C.I.A.R.I), Port Blair, India; ^2^Department of Veterinary Microbiology, West Bengal University of Animal and Fishery Sciences (W.B.U.A.F.S), Kolkata, India; ^3^Institute of Animal Hygiene and Veterinary Public Health, University of Leipzig, Leipzig, Germany; ^4^Division of Biochemistry, ICMR-Regional Medical Research Centre (I.C.M.R-R.M.R.C), Port Blair, India; ^5^Department of Veterinary Pathology, West Bengal University of Animal and Fishery Sciences (W.B.U.A.F.S), Kolkata, India

**Keywords:** Andaman and Nicobar Islands, multi-strain probiotics, backyard poultry, growth promoter, immunomodulation, serum biochemistry

## Abstract

Antibiotic growth promoters (AGPs) are widely used as feed additives to enhance the immunity, and productivity in the poultry industries. But over usage of AGPs has led to multi drug-resistance among pathogens. Nonspecific immunomodulators like probiotics have emerged as competent replacements of AGPs. Probiotics plays a key role in gut microbial health by its mechanism of action and modulation of host immune system. No prior research has been conducted in the Andaman and Nicobar Islands, India to elucidate the direct influence of probiotics on health and immunity of backyard poultry. To explore an efficient alternative to AGP, a commercial multi strain probiotics (Bifilac*^R^*) was evaluated in Vanaraja, a popular backyard poultry breed reared in the islands. 120 newly hatched Vanaraja chicks were chosen, 30 chicks were randomly allocated into 4 different groups for 60 days. For the negative control (NC), chicks were fed only basal diet. For the positive control (PC), chicks were fed with basal diet + AGP (Tetracycline). Test group (T1) was fed basal diet + 0.1% of Bifilac*^R^*. The test group (T2) was fed basal diet + 0.3% of Bifilac*^R^*. The results showed that the mean body weight of chicks supplemented with 0.1% (T1) and 0.3% (T2) of multi-strain probiotics was significantly higher (*p* ≤ 0.05) compared to the control groups. A significant increase (*p* ≤ 0.05) in FCR was also observed among T1 and T2 at different time intervals. Both T1 and T2 expressed significant changes (*p* ≤ 0.05) in biochemical parameters such as albumin, globulin, BUN, total bilirubin, SGOT and SGPT at different time intervals than the control groups. A significant decrease (*p* ≤ 0.05) was noticed in T1 and T2 groups in the levels of triglycerides, HDLc, LDLc, total cholesterol, superoxide production, lipid peroxidation at different time intervals. A significant increase (*p* ≤ 0.05) was observed in the levels of HSP70, IL4, IL2, and lymphocyte proliferation in T1 and T2 compared to the control groups. After histomorphological analysis, an increase (*p* ≤ 0.001) in villus height (μm) and crypt depth (μm) in duodenum and jejunum were noticed in T1 and T2. In short, multi-strain probiotics supplementation showed its potential as an overall growth promoter in terms of improved growth performance, favorable physiological functions, enhanced immunomodulatory effects and better intestinal morphology in a widely reared backyard poultry breed of Andaman and Nicobar Islands, India, hence can be nominated as a potential alternative to commercial antibiotics at ground level.

## Introduction

The poultry sector is known for contributing remarkably in livelihood support and ensuring nutritional security amid growing global demands. In particular, rural poultry is vital for small and resource-limited rural communities and is typically reared in extensive and semi-intensive systems, making it one of the fastest-growing segments in agriculture (Nyoni and Masika, 2012). Backyard poultry breeds are always highly acclaimed for their superior adaptability in their habitat, ability to survive in harsh climate, disease resilience, high nutritional value, also the requirement of less infrastructure set-up and low management (Gondwe and Wollny, 2002).

To meet the increasing global demand, poultry flocks often endure significant stress. As a consequence, commercial antibiotics are frequently used to prevent various avian disease, to promote growth, and boost immunity in poultry flocks (Cheng et al., 2015). However, the widespread use of antibiotics has led to the emergence of antibiotic-resistant pathogens (Garcia-Migura et al., 2014; Roth et al., 2019). World Health Organization has identified antibiotic resistance as “a serious threat to public health worldwide that requires action across all government sectors and society” (WHO, 2015). In response, several countries such as Sweden, Denmark, South Korea, Germany, and Taiwan have banned the use of antibiotics in animal feed (Ziggers, 2011). Also, nations like Netherlands, France, Italy, Belgium, Norway, and Finland have established national programs to monitor antimicrobial resistance (Garcia-Migura et al., 2014).

Over an extended period, studies have highlighted the potentials of probiotics and their ability to curb antibiotic resistance in pathogenic bacteria within the poultry sector. In recent years, non-specific immunomodulators like prebiotics, probiotics, postbiotics, synbiotics, essential oils, polysaccharides, enzymes, and organic acids have emerged as remarkably effective substitute for marketed antibiotics, demonstrating safety, feasibility to strengthen the poultry microbiota (Callaway et al., 2017; Shi et al., 2019). The word “probiotics” originated from Greek language, which means “*prolife*” (Shokryazdan et al., 2017). FAO/WHO illustrated probiotics as “live organisms that when administered in adequate amount confer a health benefit on the host” (Food Agriculture Organization/World Health Organization, 2001). Probiotics are recognized for their capacity to enhancing the microflora and bolstering immunity (Chen et al., 2017). Integrating probiotics into animal diets has upgraded their growth and productivity outcomes, digestibility, immunity, fecal microflora etc. in livestock (Cavalheiro et al., 2015; Zhao and Kim, 2015; Lan et al., 2017). The action mechanism of probiotics primarily involves outcompeting harmful pathogens. This includes producing inhibitory substances, blocking pathogen adhesion, competing for available nutrients, reducing toxin bioavailability, and influencing the host’s immune response (Hernandez-Patlan et al., 2020).

Multi-strain probiotics are combinations of different strains from the same species or various bacterial genera, which support the host’s health and immune system (Kwoji et al., 2021). An increase in the chicken body weight was observed when enriched with a *Lactobacillus-*based probiotics at a concentration of 1 × 10^9^CFU/g, either alone or in combination with prebiotics or synbiotics (Mookiah et al., 2014). *Pasteurella multocida* challenged broilers showed improved growth efficiency, feed consumption, and intestinal wellbeing when enriched with multi-strain probiotics blend containing *L. fermentum, S. cerevisiae, E. faecium, L. plantarum*, and *P. acidilactici* (Lambo et al., 2021). Earlier reports showed the positive effects of multi-strain probiotics supplementation on growth efficiency, lipid oxidation, intestinal structure, pathogen control, and gut microbiome development in chickens (Kazemi et al., 2019; Ramlucken et al., 2020; Fesseha et al., 2021).

The usage of probiotics in rural backyard poultry is still an unexplored area of study, especially in tropical islands. Hence, to assess the benefits and potentials of multi-strain probiotics on rural poultry, this study is conceptualized. The present study sheds light on the impact of multi-strain probiotics on the growth outcomes, biochemical serum profile, immunomodulation, and intestinal architecture of a rural poultry, viz. Vanaraja, a dual-purpose chicken variety, under the tropical climate of the Andaman and Nicobar Islands, India.

## Materials and methods

### Study area

**FIGURE 1:**
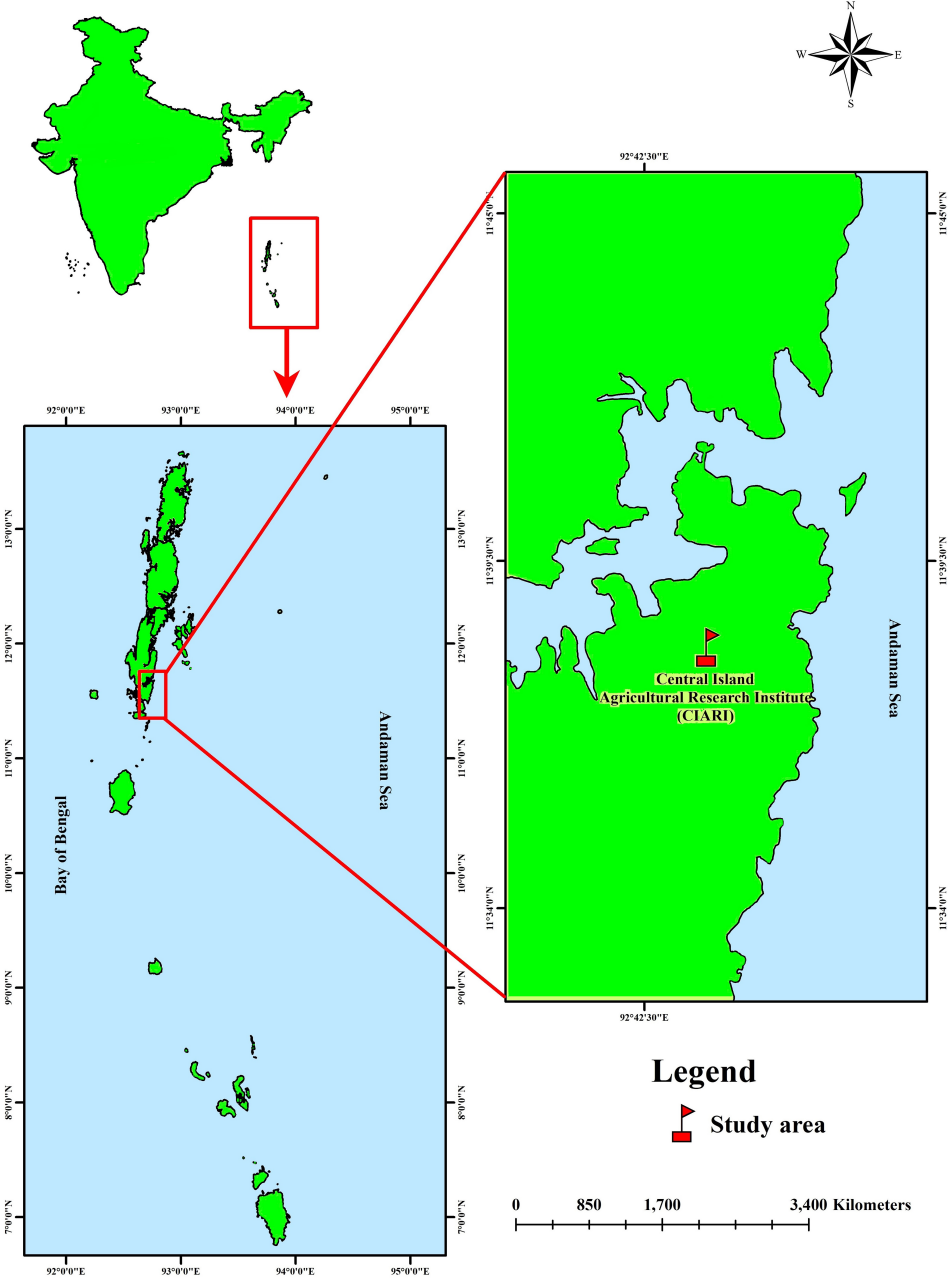
Map showing the area of the present study (C.I.A.R.I, Port Blair, Andaman and Nicobar Islands, India).

The study was conducted in the livestock farm of I.C.A.R – Central Island Agricultural Research Institute (C.I.A.R.I), Port Blair, South Andaman, India (11.6060°N, 92.7058°E) (Figure 1). Open-source software QGIS 3.16.0v “Hannover” software was used to develop the map.

### Ethical approval

Approval was obtained in accordance with F. No: AS/IAEC/22, from Institute Animal Ethical Committee, Animal House Facility of Establishment (247/GO/RBi/SL/2000/CPCSEA), ICAR-CIARI, Port Blair, Andaman and Nicobar Islands, India.

### Recording environmental data

Key environmental parameters—temperature, rainfall, humidity, and temperature-humidity index (THI) were monitored daily throughout the experiment (Table 1). THI was calculated using the formula: *THI* = 0.8 *T + RH * (T-14.4) + *46.4*, where *T* = dry-bulb temperature in°C and *RH* = relative humidity expressed as a proportion, i.e., 65% humidity is expressed as 0.65 (Habeeb et al., 2018).

**TABLE 1:** Climatic parameters during the trial.

Weeks	Avg. Temperature (°C)		Avg. Humidity (%)	Total rainfall (mm)	THI
	Max.	Min.			
1st week	32.58 ± 1.08	26.18 ± 1.27	73.57 ± 8.32	42.9 ± 10.42	85.74
2nd week	33.30 ± 1.34	25.79 ± 0.51	73 ± 5.37	62.9 ± 13.75	86.84
3rd week	31.59 ± 2.06	25.36 ± 1.55	86.75 ± 10.36	128.6 ± 21.5	86.46
4th week	28.59 ± 3.21	24.53 ± 1.06	91 ± 7.5	206 ± 25.69	82.18
5th week	32.16 ± 1.04	25.63 ± 0.67	77.71 ± 10.18	44.1 ± 10.3	85.80
6th week	30.05 ± 1.1	24.69 ± 1.6	84.88 ± 7.32	187.2 ± 28.05	83.59
7th week	31.26 ± 1.5	26.21 ± 1.13	78 ± 3.51	40 ± 9.96	84.56
8th week	31.1 ± 0.91	24.41 ± 1.27	82.43 ± 6.8	43.6 ± 7.11	84.97

All values are represented as mean ± standard deviation

### Source of probiotics

The commercially available multi-strain probiotics – *Bifilac^R^* (Tablets India Ltd., India) was procured from a commercial medical supplier. Composition of *Bifilac^R^*: *Lactobacillus sporogenes* (50M), *Streptococcus faecalis* T-110 JPC (30M), *Bacillus mesentericus* TO-A JPC (1M), *Clostridium butyricum* TO-A (2M) and excipients (q.s.).

The viability of the microorganisms present in the multi-strain probiotic “*Bifilac^R^*” and their content per gram of the product (CFU/g) were evaluated in the laboratory through standard procedures. The concentration (CFU/g) of all four microorganisms was found to be equivalent to the claims made by the commercial company. Moreover, after incorporation in the basal diet, viability of the microorganisms in “*Bifilac^R^*” was checked to ensure similar experimental conditions throughout the trial period.

### Feed preparation

The basal diet was developed as per ICAR-2013 feed standard which provides the growing chicks with essential nutrients required for ideal growth and performance. The details of the diet composition and nutrient profile are presented in Table 2.

**TABLE 2:** Ingredients of the basal diet.

Ingredients	Starter (%)	Grower (%)
Maize	37.8	38.3
Rice bran	29	31.4
Soya meal	30	27.2
Shell grit	0.6	0.6
Vitamin premix^a^	0.25	0.25
Mineral premix^b^	1	1
Lysin	0.2	0.12
Methionine	0.15	0.13
Salt	1	1
Total	100	100
**Estimated value (%)**		
Crude protein,%	22.44	20.12
P,%	1.49	1.03
Ca,%	6.4	5.9
Fiber,%	6.15	8.45
Lipid,%	12.3	14
Ash,%	1.12	1.65
Moisture,%	2.7	2.6
Carbs,%	66.86	52.02
Metabolizable energy (ME) (kcal/kg)	2519.4	2509.1

^a^Premix contained/kg of diet: Vitamin D_3_ 7 MIU, Vitamin A 25 MIU, Vitamin K_3_ 2 g, Vitamin E 16 g, Vitamin B_2_ 10 g, Vitamin B_1_ 2 g, Vitamin B_12_ 30 mg, Vitamin B_6_ 3 g, nicotinic acid 24 g, Pantothenic acid 16 g, Folic acid 2 g, biotin 50 mg.

^b^Premix contained/kg of diet: Cu 1,200 mg, Co 150 mg, Mg 6,000 mg, I 325 mg, Na 5.9 mg, Fe 1,500 mg, K 100 mg, Mn 1,500 mg, Zn 9,600 mg.

### Experimental animals

120 newly hatched Vanaraja chicks (sex ratio—1:1) were purchased from a commercial brooding facility (Amman Poultry and Feeds, Port Blair, A & N Islands, India). Each chick was individually weighed and randomly assigned to 4 groups of 30 chicks each.

• Group – I (Negative Control): This group was provided with only basal diet (*n* = 30).
• Group – II (Positive Control): This group was provided with basal diet supplemented with AGP (*n* = 30). AGP used was Tetracycline (200 g/ton)
• Group – III (T1): This group was provided with basal diet supplemented with 0.1% multi-strain probiotics (*n* = 30).
• Group – IV (T2): This group was provided with basal diet supplemented with 0.3% multi-strain probiotics (*n* = 30).

The farms were cleaned and disinfected thoroughly in advance. The light and dark periods were maintained according to the standard guidelines. The micro temperature of the farm was noted on daily basis. The chicks were raised in a deep litter system. Mortality was continuously monitored and recorded. Throughout the 60-day trial period, both feed and water were provided *ad libitum*.

### Collection of samples

Blood was collected from individual chicken on Day 0 and 15-day intervals thereafter irrespective of the sex of the poultry birds up to the conclusion of the experiment. Following standard aseptic procedures, whole blood (1.5–2 mL) was drawn from the wing vein using syringe and transferred into vacutainers preloaded with clot activators (J.K. Diagnostics, Rajkot, India). The samples were left undisturbed at room temperature for 1 h before being centrifuged at 3,000 g for 10 min at 25°C.

### Growth parameters

The body weight of each individual bird was measured on Day 0 and at 15-day intervals thereafter. Daily feed consumption of each group was recorded on regular basis. The daily feed intake for each group was determined by the subtracting the initial quantity of feed given from the final residual feed. For each trial group, the feed conversion ratio (FCR), average daily gain (ADG), and average daily feed intake (ADFI) were also calculated at 15-day intervals.

### Estimation of serum biochemical parameters

The biochemical parameters such as albumin, total protein (TP) (ARKRAY Healthcare Pvt. Ltd), creatinine, lactate dehydrogenase (LDH), glucose, total bilirubin (TB), alkaline phosphatase (ALP), serum glutamic pyruvic transaminase (SGPT), serum glutamic oxaloacetic transaminase (SGOT) (OPTIMUS, Madurai, India), and blood urea nitrogen (BUN) (ERBA Diagnostics, Mannheim GmbH, Germany) were analyzed by commercially available kits with the manufacturer’s protocol. Globulin (G) was determined by subtracting the serum protein level from the serum albumin level (Total Protein –Albumin = Globulin) (Busher, 1990).

The lipid profile such as high-density lipoprotein cholesterol (HDLc), low-density lipoprotein cholesterol (LDLc), total cholesterol (TC), and triglycerides (TG), (OPTIMUS, Madurai, India) were analyzed using commercialized kits with the manufacturer’s protocol.

Antioxidant properties in serum were determined bycolorimetry (Antioxidant Activity Estimation kit, HiMedia Laboratories Pvt. Ltd., Nashik, India). Lipid peroxidation was estimated bya colorimetric kit (TBARS estimation kit for lipid peroxidation, HiMedia Laboratories Pvt. Ltd., Nashik, India) and nitric oxide was analyzed using commercial kit (HiMedia Pvt Ltd., Nashik, India), all performed as recommended by the manufacturer.

### Estimation of immune parameters

IL6, IL4, IL2, HSP70, INF-γ, TLR4 responses were determined by using commercially available ELISA kits (Life Technologies Pvt Ltd., Delhi, India). *In vitro* oxidative radical production by neutrophils were measured by nitro blue tetrazolium (NBT) assay (Siwicki et al., 1998). Blood lymphocyte proliferation was assessed by *in vitro* lymphoproliferation assay (Daly et al., 1995).

### Histomorphological analysis

A total of 12 poultry birds, three representative chicks from each trial group were humanely euthanized via cervical dislocation after the completion of experimental trials. Visceral samples were preserved in 10% Formalin till further processing. The samples were then processed for standard histomorphological analysis (Biswas et al., 2022). Respective slides of intestinal sections were prepared to measure villus height and crypt depth. The measurement for crypt depth and villi height were carried out using Olympus CX41RF Microscope (Olympus Corporation, Tokyo, Japan) at 40X magnification. Images of crypts and villus were captured via an industrial digital camera (Panasonic CMOS sensor) connected to a computer. The measurements were made using the imaging software –Image View.

### Statistical analysis

Data are expressed as Mean ± Standard deviation (SD). Statistical significance was assessed by analysis of variance (ANOVA) following Bonferroni *post hoc* and *t*-test was performed with GraphPad Prism 9.3.1 software, considering the *p* ≤ 0.05 as significant.

## Results

### Growth performance

Multi-strain probiotics (*Bifilac^R^*) supplementation significantly increased (*p* ≤ 0.05) the average daily weight gain (ADG) in groups T1 (0.1%) and T2 (0.3%) compared to the NC and PC groups during days 45–60 of the trial. A significant improvement (*p* ≤ 0.05) in the FCR was also observed in the T1 and T2 groups during days 30–45 and 45–60 of the experiment compared to the NC group. The PC group also showed a significant increase in FCR when compared to the NC and T2 groups during days 30–45 and 45–60 of the experiment. In summary, the addition of multi-strain probiotics to the diet positively influenced growth performance and feed utilization in growing chicks (Table 3).

**TABLE 3:** Growth traits of Vanaraja following multi-strain probiotics supplementation.

Attributes	Groups				*p-values*
ADFI (g)	NC	PC	T1	T2	
(0–15 d)	150.5 ± 80.79	152.27 ± 85.25	149.60 ± 87.59	147.03 ± 89.94	0.932
(15–30 d)	328.4 ± 77.94	326.87 ± 74.99	329.63 ± 80.35	326.73 ± 72.99	0.981
(30–45 d)	894.27^a^ ± 459.8	865.67^b^ ± 475.33	855.37^b^ ± 465.58	855.47^b^ ± 468.71	0.003
(45–60 d)	1574^a^ ± 405.72	1565.43^ab^ ± 420.8	1548.37^b^ ± 414.56	1549.47^b^ ± 419.82	0.022
**Attributes**	**Groups**				***p***
**ADG (g)**	**NC**	**PC**	**T1**	**T2**	
(0–15 d)	124.63^a^ ± 19.59	108.43^b^ ± 22.48	131.33^a^ ± 18.47	100.47^b^ ± 28.46	< 0.001
(15–30 d)	280.3^b^ ± 61.45	292.53^b^ ± 14.58	324.03^a^ ± 54.58	298.37^ab^ ± 69.69	0.019
(30–45 d)	540.3^a^ ± 64.66	492.1^b^ ± 95.83	484.57^b^ ± 95.04	547.27^a^ ± 57.9	0.003
(45–60 d)	791.17^c^ ± 64.91	883.37^b^ ± 45.97	864.33^b^ ± 47.07	914.33^a^ ± 17.11	< 0.001
**Attributes**	**Groups**				***p***
**FCR**	**NC**	**PC**	**T1**	**T2**	
(0–15 d)	1.21^b^ ± 0.1	1.4^a^ ± 0.1	1.14^b^ ± 0.1	1.46^a^ ± 0.1	0.007
(15–30 d)	1.17^a^ ± 0.04	1.02^ab^ ± 0.04	1.02^c^ ± 0.03	1.09^b^ ± 0.04	0.004
(30–45 d)	1.66^b^ ± 0.02	1.76^a^ ± 0.02	1.77^a^ ± 0.03	1.56^c^ ± 0.02	< 0.001
(45–60 d)	1.99^a^ ± 0.01	1.77^b^ ± 0.01	1.79^b^ ± 0.01	1.69^c^ ± 0.02	< 0.001

*All values are represented as mean ± standard deviation. Different alphabets of a row in superscript indicate significant differences.

### Biochemical profile

**FIGURE 2:**
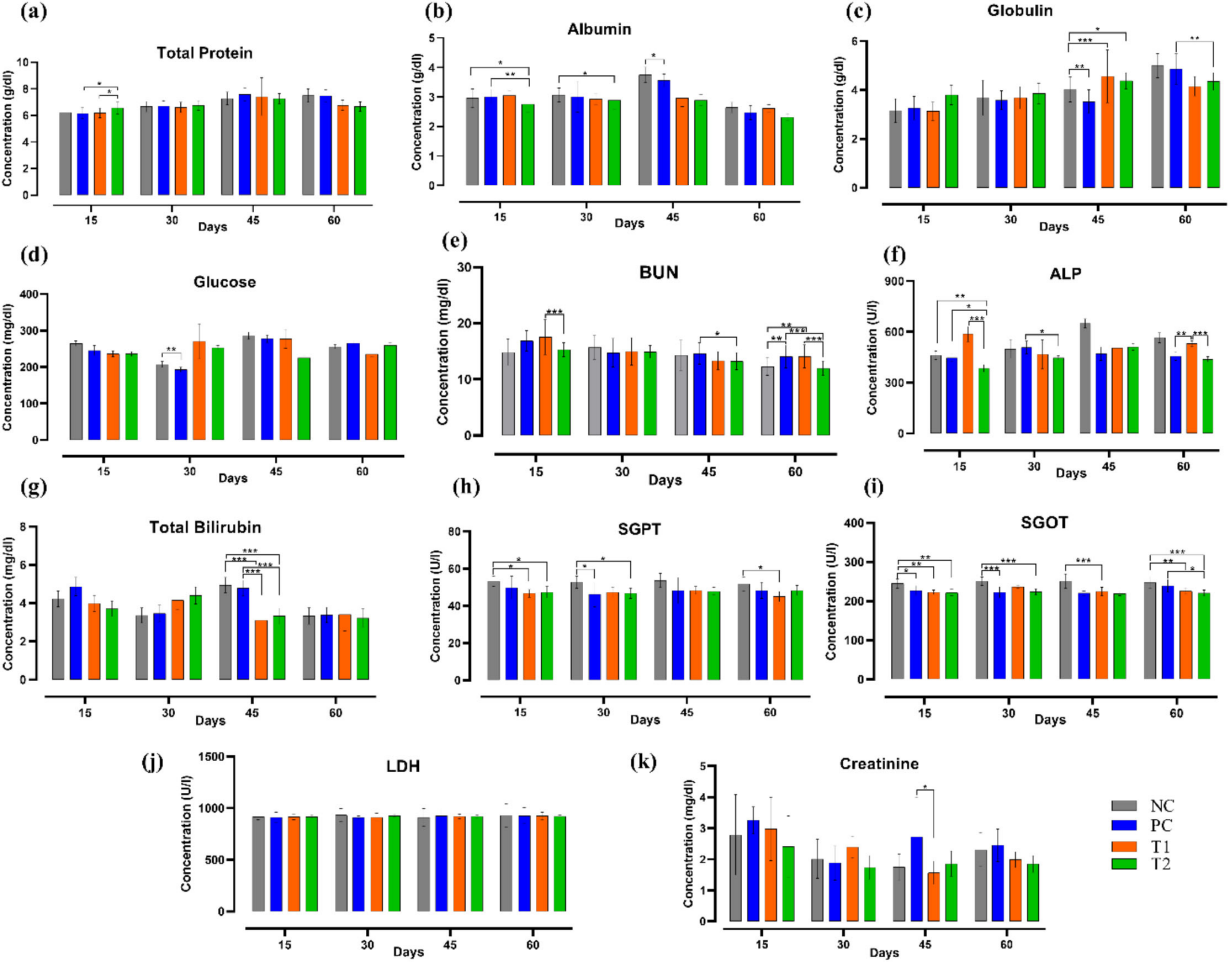
Effect of multi-strain probiotics supplementation on the biochemical profile. **(a)** Total protein, **(b)** albumin, **(c)** globulin, **(d)** glucose, **(e)** blood urea nitrogen (BUN), **(f)** alkaline phosphatase (ALP), **(g)** total bilirubin, **(h)** serum glutamate pyruvate transaminase (SGPT), **(i)** serum glutamate oxaloacetate transaminase (SGOT), **(j)** lactate dehydrogenase (LDH), and (k) creatinine. Significance levels are *p ≤ 0.05; **p ≤ 0.01; ***p ≤ 0.001.

Following multi-strain probiotics (*Bifilac^R^*) supplementation, on the 15th and 30th day of the experiment, a significant decrease (*p* ≤ 0.05) in albumin (g/dL) level was observed in the T2 group relative to the control groups NC and PC. The level of globulin (g/dL) on the 45th day was significantly high in T1 (*p* ≤ 0.001) and T2 (*p* ≤ 0.05) groups when compared to NC. On the 60th day, the level of globulin (g/dL) was significantly reduced (*p* ≤ 0.01) in T2 than that of PC group. Blood urea nitrogen (BUN) (mg/dL) level was observed to be significantly decreased (*p* ≤ 0.05) in the T2 group than the PC group on the 45th day. BUN levels were significantly different (*p* ≤ 0.01) on 60th day among the T1 and T2 groups relative to the control groups NC and PC. A significant difference (*p* ≤ 0.05) was observed in ALP (U/L) level throughout the experimental trial among the treated groups and the control groups. Total bilirubin (mg/dL) levels were significantly different (*p* ≤ 0.001) on the 45th day of the trial among the treated groups T1 and T2 when compared to the control groups NC and PC. SGPT (U/L) level in T1 and T2 group showed significant decrease (*p* ≤ 0.05) on 15th, 30th, and 60th day of experiment when compared to the control groups NC and PC. SGOT (U/L) level has notably differed among the treated groups T1 and T2 relative to the control groups NC and PC throughout the experiment. Creatinine (mg/dL) level was significantly different (*p* ≤ 0.05) in the T1 group than the PC group on 45th day of the experiment. In brief, multi-strain probiotics (*Bifilac^R^*) supplementation has considerably influenced the biochemical profile (Figure 2).

### Lipid profile

**FIGURE 3:**
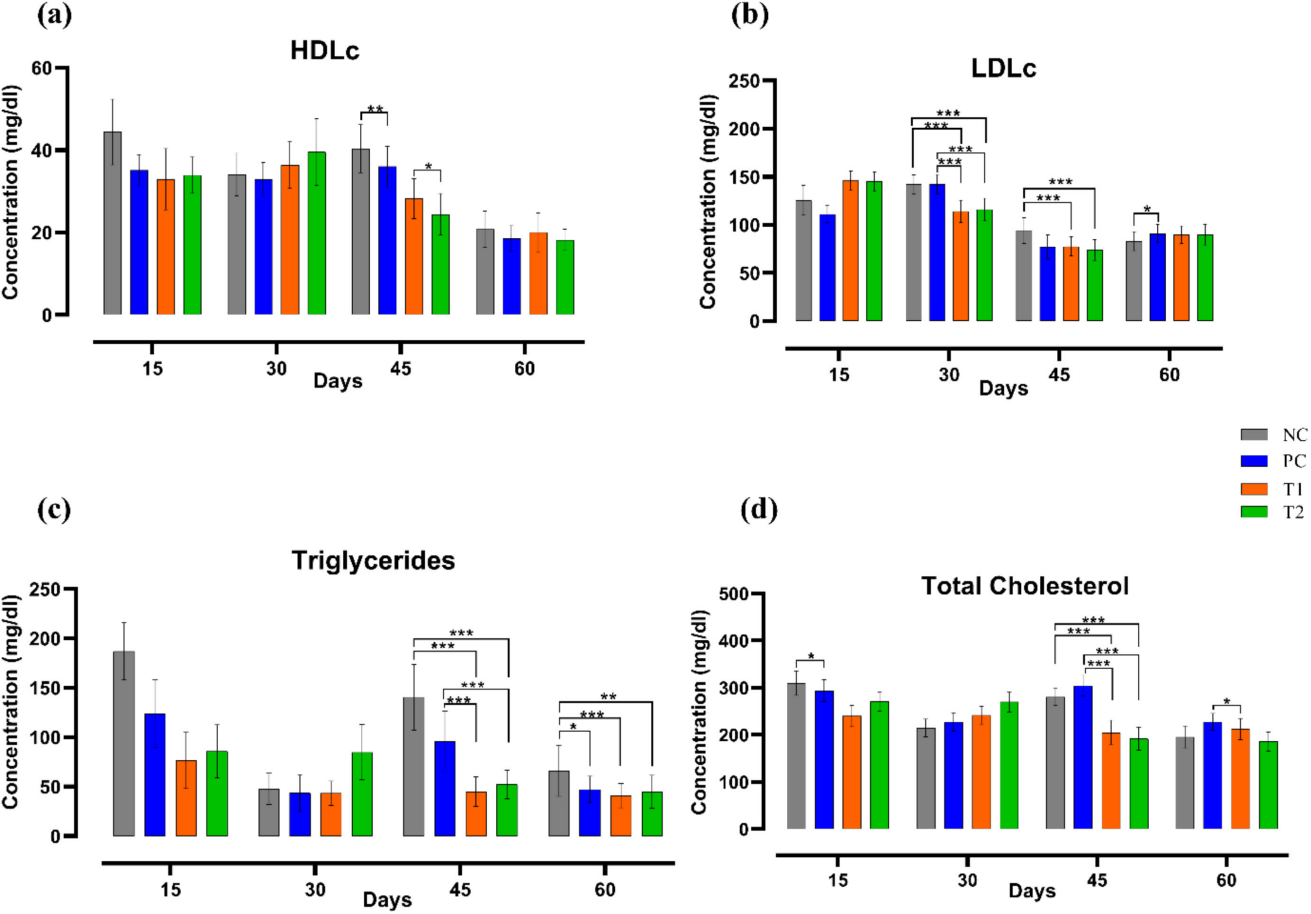
Effect of multi-strain probiotics supplementation on the lipid profile. **(a)** HDLc, **(b)** LDLc, **(c)** triglycerides, and **(d)** total cholesterol. Significance levels are *p ≤ 0.05; **p ≤ 0.01; ***p ≤ 0.001.

After multi-strain probiotics supplementation, LDLc (mg/dl) levels in the T1 and T2 groups were reduced significantly (*p* ≤ 0.001) on 30th, 45th day of the experiment when compared to the control groups NC and PC. Triglyceride (mg/dL) level was significantly decreased (*p* ≤ 0.001) in T1 and T2 groups relative to the control groups NC and PC on the 45th day of trial. On the 60th day, triglyceride (mg/dL) level was significantly reduced in T1 (*p* ≤ 0.001) and T2 (*p* ≤ 0.01) when compared to NC group. Total cholesterol (mg/dL) level was significantly reduced (*p* ≤ 0.001) in T1 and T2 groups when compared to the control groups NC and PC on the 45th day of the experiment. On the 60th day, total cholesterol (mg/dL) level in T1 was significantly decreased (*p* ≤ 0.05) relative to the PC group. In summary, multi-strain probiotics (*Bifilac^R^*) supplementation has strongly affected the lipid profile (Figure 3).

### Antioxidant profile

**FIGURE 4:**
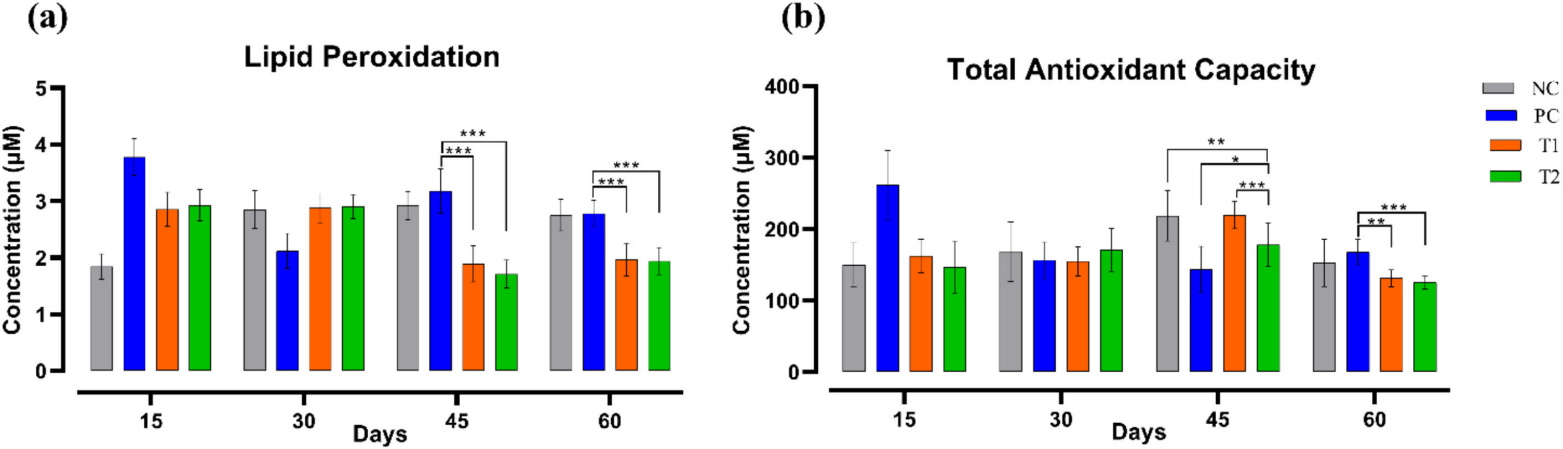
Effect of multi-strain probiotics supplementation on the antioxidant profile. **(a)** Lipid peroxidation, and **(b)** total antioxidant capacity. Significance levels are *p ≤ 0.05; **p ≤ 0.01; ***p ≤ 0.001.

Lipid peroxidation (μM) level was significantly decreased (*p* ≤ 0.001) in T1 and T2 when compared to the PC group on the 45th and 60th day of the experiment after multi-strain probiotics supplementation. The total antioxidant capacity (μM) after the supplementation was notably differed throughout the trial in the T1 and T2 groups relative to the control groups NC and PC. To summarize, multi-strain probiotics (*Bifilac^R^*) supplementation has substantially impacted the antioxidant profile of Vanaraja breed (Figure 4).

### Serum cytokine profile

**FIGURE 5:**
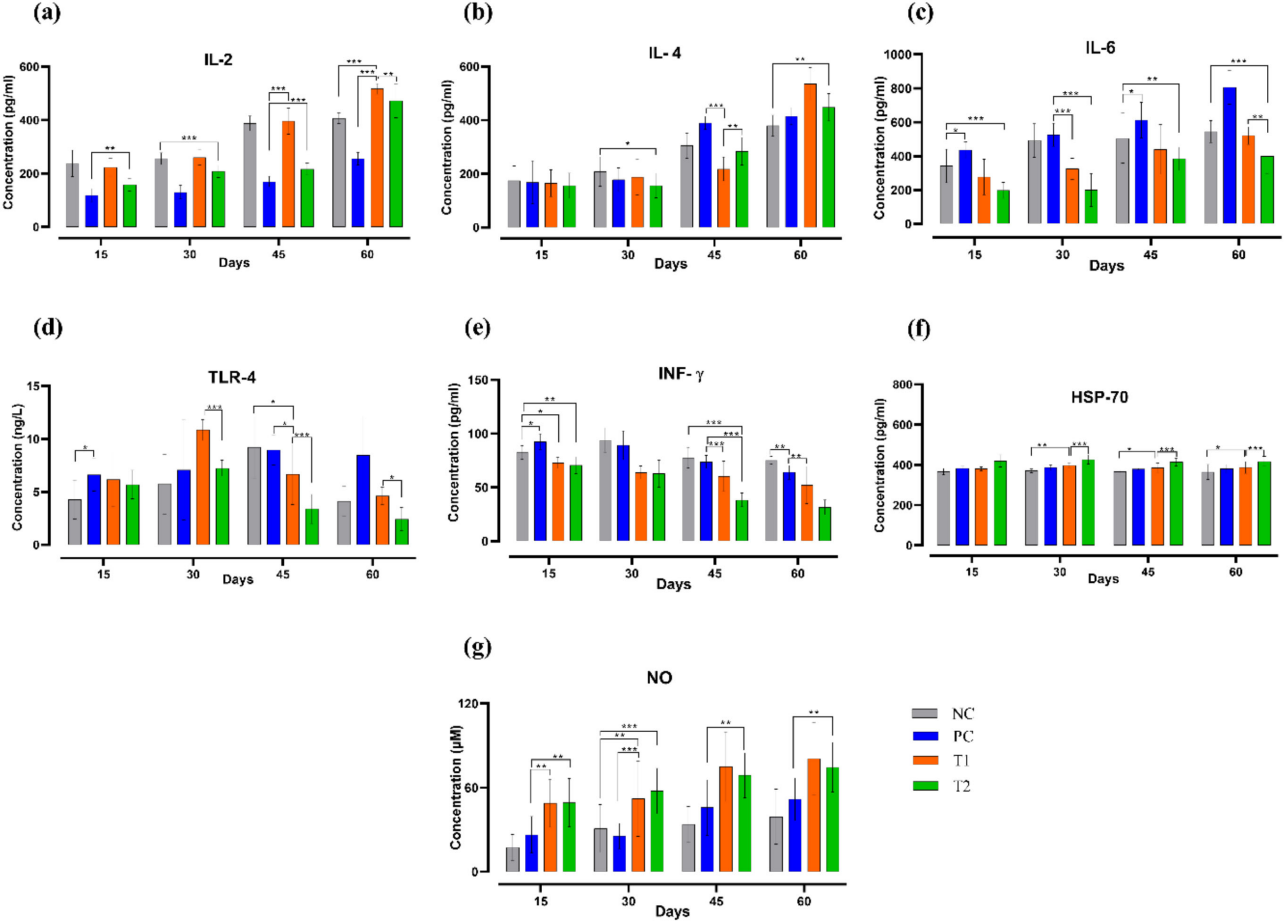
Effect of multi-strain probiotics supplementation on the serum cytokines. **(a)** IL2, **(b)** IL4, **(c)** IL6, **(d)** TLR4, **(e)** IFN-γ, **(f)** HSP70, and **(g)** Nitric Oxide (NO). Significance levels are *p ≤ 0.05; **p ≤ 0.01; ***p ≤ 0.001.

After multi-strain probiotics supplementation, on the 15th day, the concentration of IL2 (pg/mL) in the T2 group showed significant upregulation (*p* ≤ 0.01) compared to the PC group. On the 30th day, the T2 group again showed significant upregulation (*p* ≤ 0.001) in the concentration of IL2 (pg/mL) than the NC group. On the 45th day, both T1 and T2 groups showed significant upregulation (*p* ≤ 0.001) in the concentration of IL2 (pg/mL) than the PC group. On the 60th day, the T1 group showed significant upregulation (*p* ≤ 0.001) in the concentration of IL2 (pg/mL) than the control groups NC and PC. After multi-strain probiotics supplementation, IL4 (pg/mL) concentrations in T1 and T2 were notably upregulated compared to the control groups throughout the experiment. Concentrations of IL6 (pg/mL), IFN-γ (pg/mL), and TLR4 (ng/L) were remarkably downregulated throughout the trial in the T1 and T2 groups relative to the control groups NC and PC. A significant increase (*p* ≤ 0.05) in HSP70 (pg/mL) concentration was observed after the supplementation in the T1 group when compared to the NC group. A significant increase (*p* ≤ 0.0) in NO (μM) concentration was also observed throughout the experiment in the T1 and T2 relative to the control groups NC and PC. In short, multi-strain probiotics (*Bifilac^R^*) administration has highly influenced the serum cytokines (Figure 5).

### Immune parameters

**FIGURE 6:**
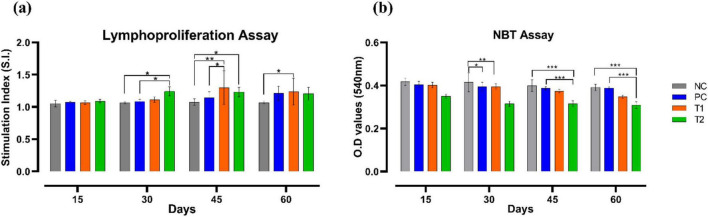
Effect of multi-strain probiotics supplementation on the immune parameters. **(a)** Lymphoproliferation assay, and **(b)** NBT reduction assay. Significance levels are *p ≤ 0.05; **p ≤ 0.01; ***p ≤ 0.001.

Lymphoproliferation assay showed apparent elevation in the number of lymphocytes in different interval of time. On the 30th day, the T2 group showed significant increase (*p* ≤ 0.05) in the number of lymphocytes than the control groups NC and PC. On the 45th day, the T1 group showed significant surge in lymphocytes proliferation than the control groups NC (*p* ≤ 0.01) and PC (*p* ≤ 0.05). On the 45th day, the T2 group also showed significant increase (*p* ≤ 0.05) in lymphocytes proliferation than the NC group. On the 60th day, the T1 group showed significant increase (*p* ≤ 0.05) in lymphocytes proliferation than the NC group. NBT reduction assay showed considerable reduction in superoxide production in treated groups T1 and T2 than that of the control groups NC and PC across the trial. On 30th day, T1 group showed significant reduction (*p* ≤ 0.01) in superoxide production than the NC group. On 45th day, T2 group also showed significant decrease (*p* ≤ 0.001) in superoxide production when compared to both the control groups NC and PC. Similarly, on 60th day, T2 group showed significant decline (*p* ≤ 0.001) in superoxide production than the control groups NC and PC. To sum up, multi-strain probiotics (*Bifilac^R^*) administration has noticeably modulated the immune parameters when given in various concentrations (Figure 6).

### Histomorphological analysis

**FIGURE 7:**
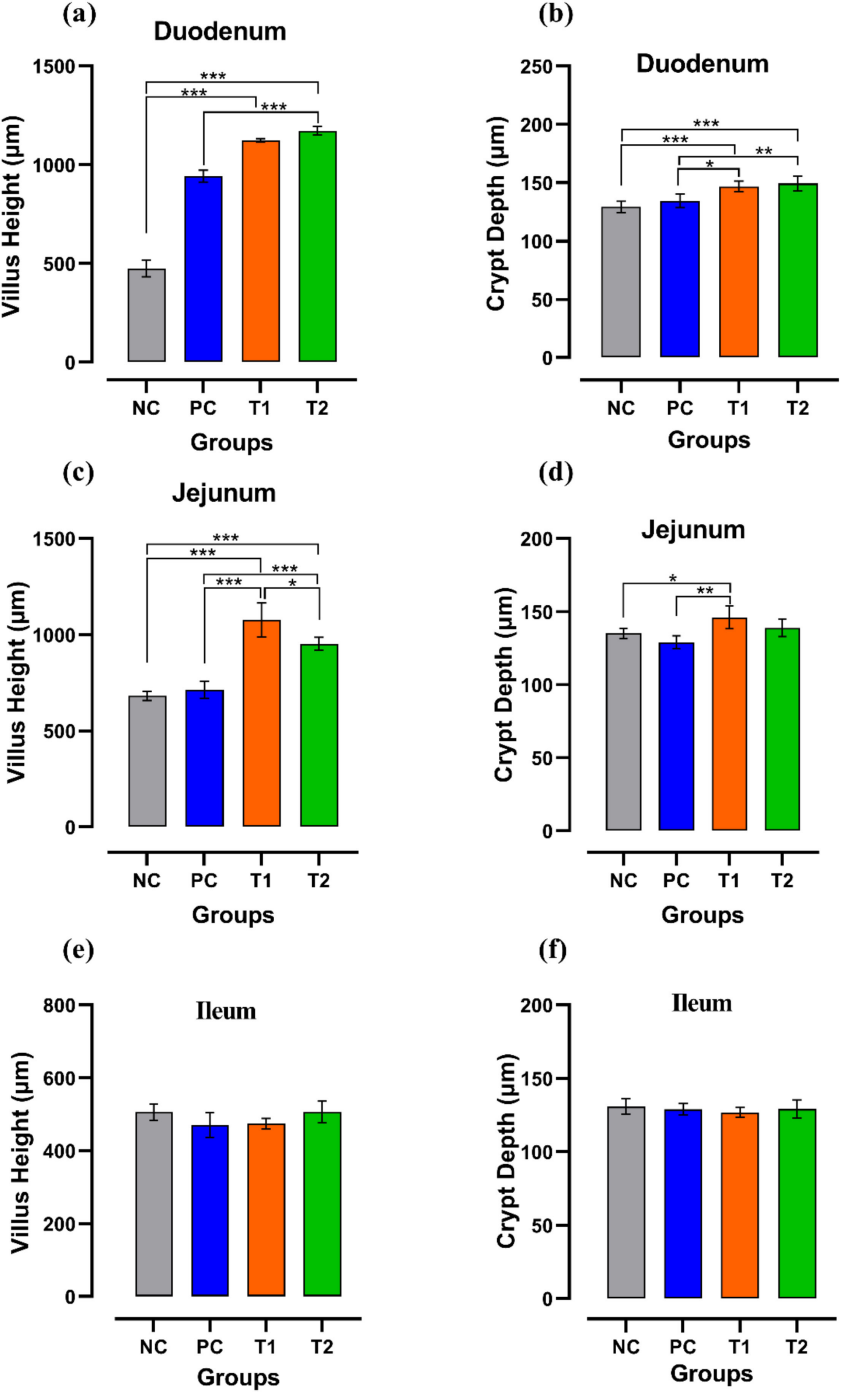
Effect of multi-strain probiotics supplementation: in duodenum. **(a)** Villus height (μm) **(b)** crypt depth (μm); in jejunum—**(c)** villus height (μm) **(d)** crypt depth (μm); and in ileum—**(e)** villus height (μm) **(f)** crypt depth (μm). Significance levels are *p ≤ 0.05; **p ≤ 0.01; ***p ≤ 0.001.

**FIGURE 8:**
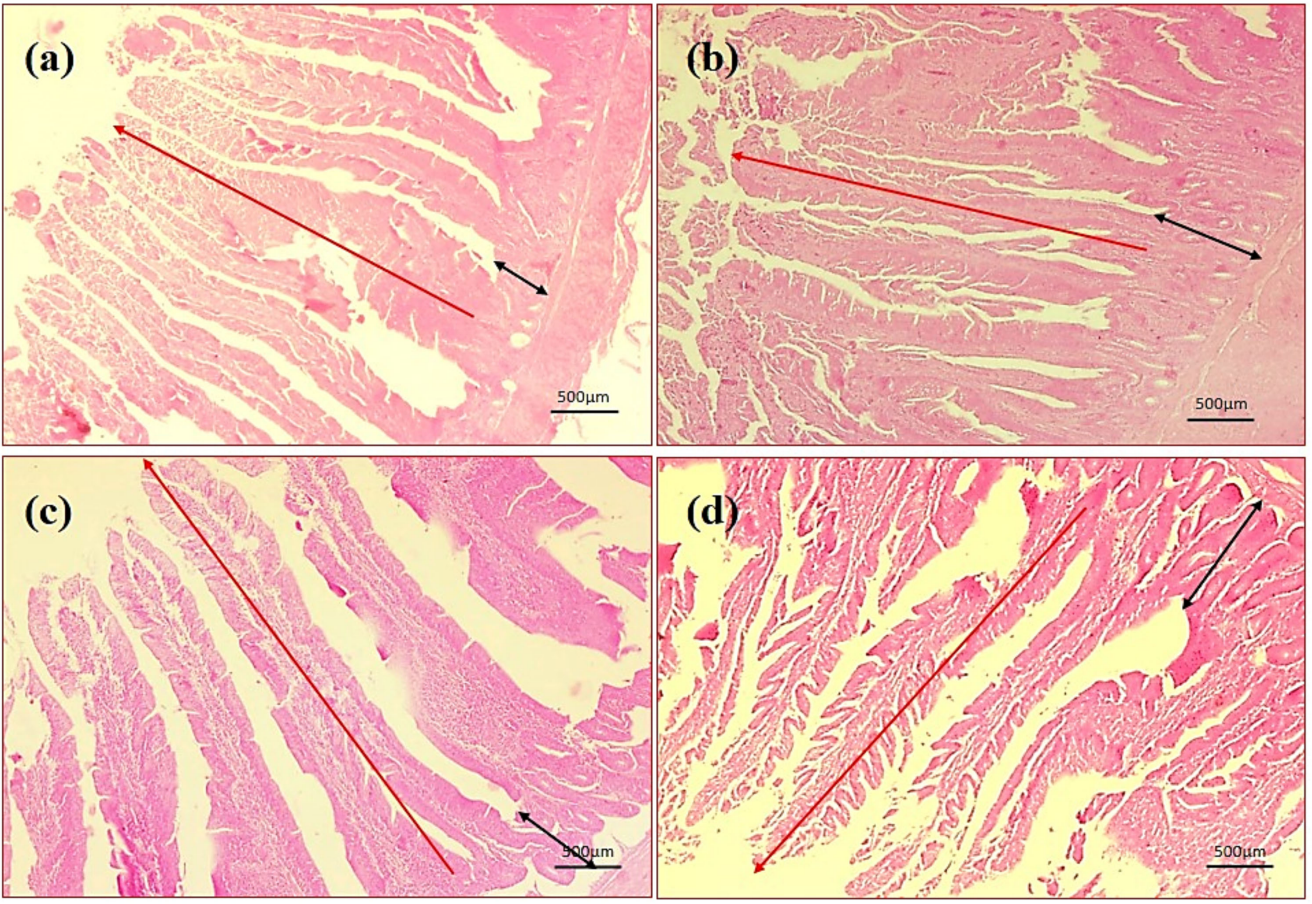
Photomicrography of duodenum (40X magnification). **(a)** Negative control (NC), **(b)** positive control (PC), **(c)** T1 supplemented with 0.1% of multi-strain probiotics, and **(d)** T2 supplemented with 0.3% of multi-strain probiotics.

**FIGURE 9:**
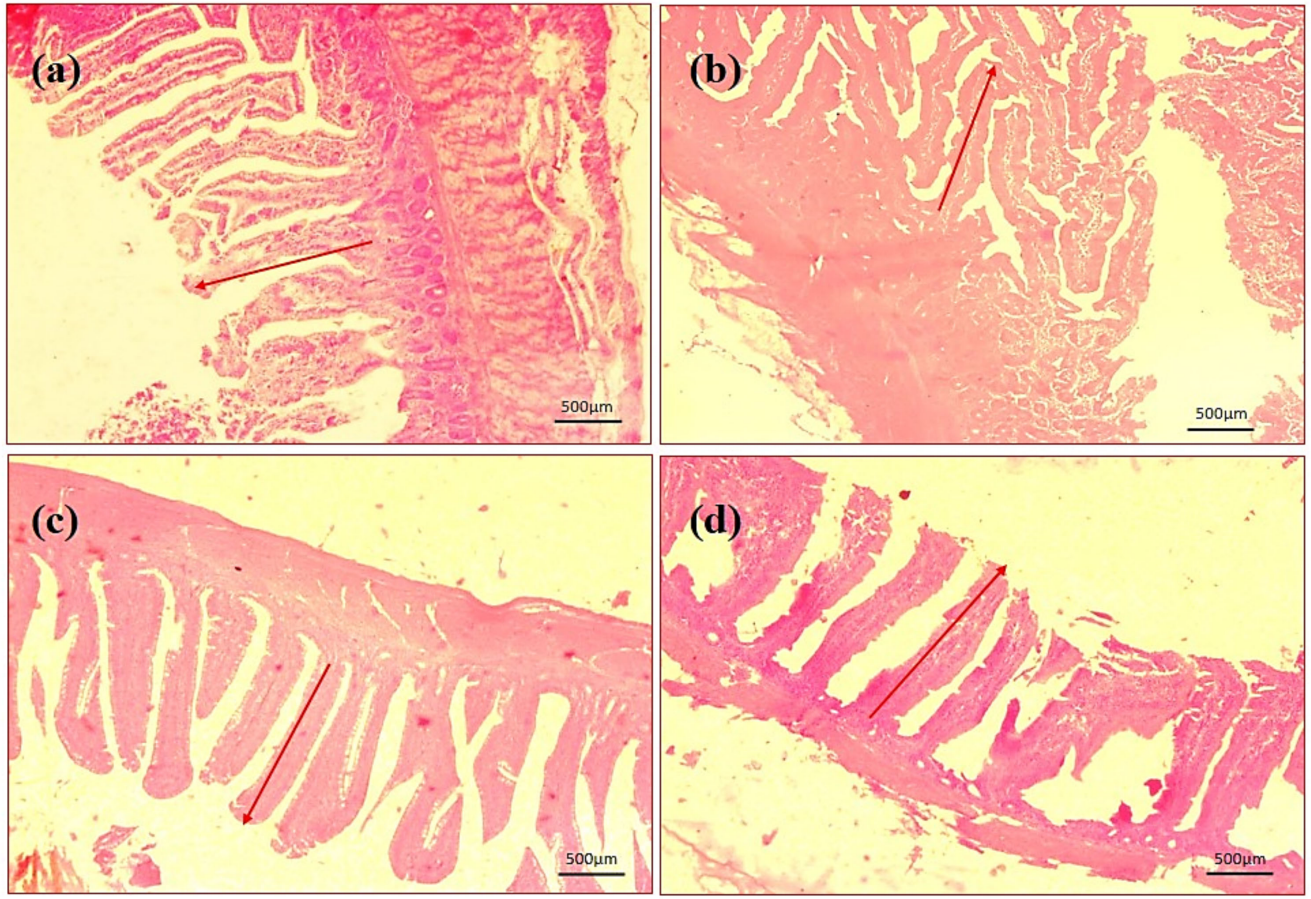
Photomicrography of jejunum (40X magnification). **(a)** Negative control (NC), **(b)** positive control (PC), **(c)** T1 supplemented with 0.1% of multi-strain probiotics, and **(d)** T2 supplemented with 0.3% of multi-strain probiotics.

**FIGURE 10:**
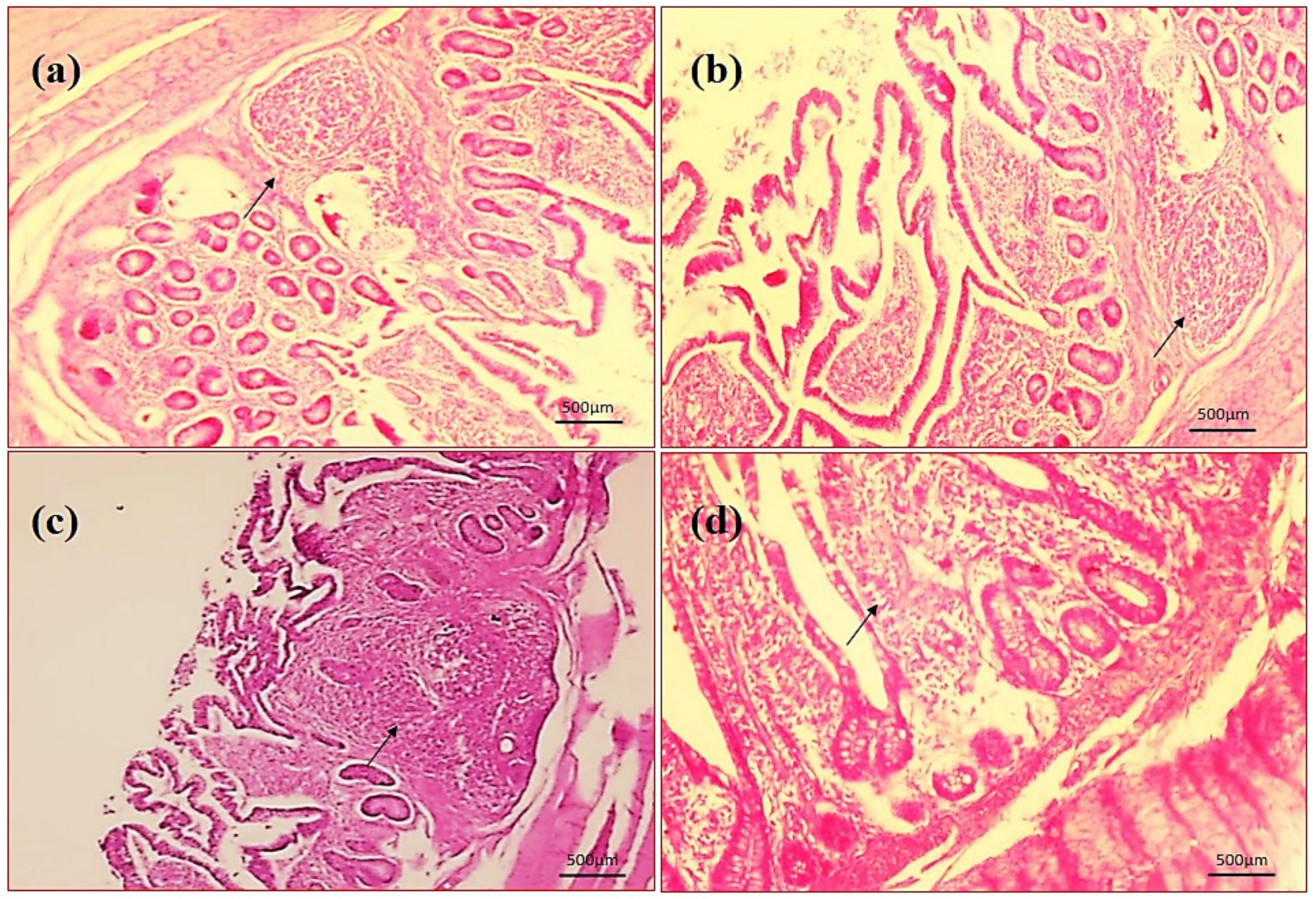
Photomicrography of ileum (40X magnification). **(a)** Negative control (NC), **(b)** positive control (PC), **(c)** T1 supplemented with 0.1% of multi-strain probiotics, and **(d)** T2 supplemented with 0.3% of multi-strain probiotics.

By assessing the influence of multi-strain probiotics on the histological parameters of intestine, it was observed that the villi height (μm) and crypt depth (μm) in duodenum was significantly greater (*p* ≤ 0.001) in the T2 group than the control groups NC and PC (Figure 7). In the jejunum, the height of villi (μm) and crypt depth (μm) was considerably more (*p* ≤ 0.05) in the T1 group than the other groups T2, NC and PC (Figure 7). In ileum, no significant changes were observed in villi height (μm) and crypt depth (μm) among any groups after the trial (Figure 7). In brief, multi-strain probiotics (*Bifilac^R^*) showed clear evidences of positive effects on chicken intestinal morphology when given in different concentrations. Photomicrographic analysis was conducted to document and evaluate histomorphological alterations across all the experimental groups, assessing the villus height and crypt depth in duodenum (Figure 8), jejunum (Figure 9), and ileum (Figure 10).

## Discussion

Probiotics represents a promising substitute for the commercial AGPs. Their supplementation has demonstrated evident influence on animal health and production performance, particularly in poultry. Findings from this study indicate that multi-strain probiotics (*Bifilac^R^*) supplementation has significantly improved feed intake, weight gain, and FCR in poultry birds. These results corroborate the findings of Li et al. (2011) and Hossain et al. (2015). Various factors can affect the feed consumption and FCR, such as type of probiotics, strain, dosage of supplementation, and the poultry breed (Zhang et al., 2021). The multi-strain probiotics intake has increased absorption of vitamins and minerals, leading to greater nutrient bioavailability and enhanced overall growth performance (Adil and Magray, 2012; Olukosi and Cowieson, 2007). The findings of this study corroborate with the results of some researchers who also observed a significant increase in BW, FI, and FCR after multi-strain probiotics supplementation in broilers (De França et al., 2023; Kalavathy et al., 2003; Neijat et al., 2019; Sobczak and Kozłowski, 2015). Multi-strain probiotics have been documented to enhance the enzymatic activity in the GI tract, which helps in the breakdown of complex materials in feed and makes them easy for absorption (Awad et al., 2009; Latorre et al., 2016). Factors affecting FCR and FI includes the feed type, feeding frequency, and host health (Musigwa et al., 2020). Several reports suggested that probiotics stimulates microbial conversion of non-digestible substances in the diet, leading to the production of short-chain fatty acids, which serve as an energy source for the host, resulting in better growth performance (Patterson and Burkholder, 2003). A nutrient-rich feed supplementation that contains the correct amount of energy and free amino acids mixed with the correct dosage of probiotics is influential for optimized feed utilization (Liang et al., 2024). Increased growth performance observed in the study after multi-strain probiotics supplementation may be due to an elevated population of good bacteria for better nutrient uptake and reduction of pathogenic microorganisms in the gut. A few contrary results indicate that multi-strain probiotics supplementation has no impact on feed intake or body weight gain in poultry birds (Anjum et al., 2005; Sirovnik et al., 2021).

Serum biochemical parameters are the indicators of the host body’s first line of defense. In this study, a noticeable surge in protein level was observed in the poultry group fed with multi-strain probiotics (*Bifilac^R^*) when compared to the control groups. This result corroborates the observations of Silva et al. (2020) and Rehman et al. (2018), who stated significant changes in total protein, albumin, and globulin levels after multi-strain probiotics supplementation in broilers. The results of the current experiment are also supported by the results of Panda et al., who found apparent changes in albumin, globulin levels, and albumin/globulin ratio in serum after probiotics supplementation, indicating a positive influence on immune responses and disease resistance (Panda et al., 2006). This change can be attributed to the competition between probiotic gut bacteria and pathogenic microbes, which limits the protein breakdown into nitrogen, thereby increasing protein and amino acid utilization (Hossain et al., 2024; Mansoub, 2010). High total protein level generally shows high protein metabolism rate (Abdel-Moneim et al., 2020; Lee et al., 2013). A report showed a notable increase in glucose, globulin, and total protein in Salmonella-infected broilers following probiotics supplementation (Abudabos et al., 2019). Some researchers have reported an increase in protein intake as the beneficial bacteria prevent degradation of protein and utilize the nitrogen of pathogens by a competitive exclusion mechanism, which results in efficient protein absorption and total serum protein (Al-Khalaifa et al., 2019; Khattab et al., 2021; Yazhini et al., 2018). A substantial increase in glucose level was noticed in the current study, which aligns with the conclusions of Abudabos et al. (2019) and Hussein (2014), who reported high glucose levels in broilers administered with probiotics. A rise in glucose level can be related to the enhancement of nutrient utilization and glycogenolysis, which leads to increased glucose absorption (Das et al., 2005). SGOT and SGPT serve as primary indicators of liver function, and increased concentrations of these indicators can cause liver damage (De et al., 2023). Reduced levels of SGOT and SGPT were observed, supporting the results of Hussein et al. (2020), who reported that probiotics administration can significantly safeguard the treated birds from hepatocellular damage compared to the control groups. Decreased levels of SGOT and SGPT showed normal liver function in chickens fed with multi-strain probiotics. This may be a valuable point to include probiotics safely as a feed additive, as it may not exert any adverse effect on liver functions in poultry. A contrary study showed no significant changes in SGOT and SGPT activity in broilers after probiotics supplementation (Lee et al., 2010). ALP is a biomarker for renal functions and is primarily derived from the epithelial cells of the bile duct, kidneys, and gut lining (Żbikowski et al., 2020). ALP activity can be elevated in chicks during the initial starter phase, but can reduce with age. This study shows a downregulation of ALP activity in chicks fed with multi-strain probiotics, which aligns with the findings of Rashidi et al. (2020) and Sharma et al. (2014). A contrary report stated that there was no significance of ALP levels after probiotic treatment in broilers (Panda et al., 2006; Sharma et al., 2014). In this study, the BUN level was decreased in the multi-strain treated groups than the control groups, which agrees with the results of various researchers who observed a decrease in the level of BUN after probiotics supplementation (El-Baky, 2013; Chen et al., 2010; Harr, 2002; Quintavalla et al., 2001). Lower levels of the BUN can be due to increased protein utilization by probiotics bacteria, leading to balanced intestinal microflora (Tang et al., 2010). BUN level is negatively related to protein deposition, and a low BUN level suggests high protein production (Yao et al., 2009). Total bilirubin level was lower in the poultry groups fed with multi-strain probiotics than in the control groups, which corroborated the results of Oladipo et al. (2023) and Udeh et al. (2020). No noticeable changes were observed in LDH and creatinine levels in the current study after probiotics supplementation, which aligns with the observations of Wu et al. (2019).

In this study, the chickens treated with multi-strain probiotics had significantly lower total cholesterol levels relative to the control groups, which matches the results of Bidura et al. (2019), Pambuka et al. (2014). Significant reduction of lipid profile in probiotics-fed broiler chickens was observed this can be due to decreased absorption and synthesis of cholesterol in the GI tract by the action of probiotics (Mohan et al., 1996). Lower levels of HDLc and LDLc were observed in the probiotics-fed chickens in the current study, which is in line with the findings of Ahmed et al. (2019) and Sun and Kim (2021). Similar other reports were also found, which state that multi-strain probiotics reduced the HDLc and LDLc levels in broilers (Panda et al., 2006; Reuben et al., 2022). Triglyceride concentration was reduced in treated groups than compared to control group in the current experiment which corroborates with the reports of Wang and Zhou (2007). Lower triglyceride levels observed may be because of the effect of downregulated lipogenesis in the liver (Alimohamadi et al., 2014; Żbikowski et al., 2020). Triglyceride reduction may be due to upregulation of hydrolysis of bile salt, which leads to less lipid absorption in the small intestine (Alkhalf et al., 2010). Few researchers also observed decreased triglycerides and total cholesterol levels in broilers supplemented with *Lactobacillus*-based probiotics (Panda et al., 2006; Mansoub, 2010). Probiotics bacteria play a crucial role in reducing or terminating the cholesterol and triglycerides synthesis in the liver by upregulating the short-chain fatty acids which subsequently downregulate the blood metabolic products (Dev et al., 2020). Kalavathy et al., reported a reduction in levels of LDLc, total cholesterol, and triglycerides in broilers when supplemented with a mixture of 12 *Lactobacillus* strains together (Kalavathy et al., 2003). A conflicting report suggests that there was no notable change in total cholesterol and triglyceride levels after multi-strain probiotics supplementation in broilers (Dev et al., 2020).

Probiotics also play an important role in influencing the oxidation state of the gut by directly showcasing the antioxidant qualities and modulating the host’s antioxidant defense signaling (Amaretti et al., 2013; Zolotukhin et al., 2018). To eliminate the free radicals in the host, the levels of *in vivo* anti-oxidant enzymes are elevated, which reflects the intensity of stress by oxidation (Rehman et al., 2018). In the current study, the level of *T*-AOC differed significantly among the trial groups but didn’t show much difference in groups treated with multi-strain probiotics. These results corroborate with the findings of Attia et al. (2013, 2018). This is probably caused due to the probiotic dosage and individual bird health. Multi-strain probiotics, especially a mixture of lactobacillus strains, can affect antioxidant enzyme activity in the host body and help in the reduction of oxidative stress damage to the intestines (Bai et al., 2018; Inatomi and Otomaru, 2018; Yu et al., 2019). Malondialdehyde (MDA) is a by-product that shows the level of lipid peroxidation. Overproduction of free radicals contributes to detrimental oxidative stress, which leads to decreased growth performances, immunosuppression, and meat quality deterioration in broilers (Salami et al., 2015; Surai, 2016). Results of this experiment showed a substantial decrease in the MDA concentration, hence a reduction in lipid peroxidation and downregulation of oxidative stress after supplementation of multi-strain probiotics to the poultry birds. These findings are consistent with the results of Zhang et al. (2021), who observed a significant reduction in MDA concentration, which led to down-regulation of lipid peroxidation in broilers administered with probiotics. Equivalent results were also described by Elbaz et al. (2023), who observed a reduction in MDA concentration in heat-stressed broilers after probiotics supplementation.

Incorporating probiotics into the broiler diet showed massive improvement of immune functions and responses (Zhen et al., 2020). In this study, up-regulation of HSP70, IL4, IL2, and down-regulation of IL6, TLR4, and INF-γ in multi-strain probiotic-fed chickens was observed. These results resemble the outcomes of Zhang et al. (2021) and Gadde et al. (2017), who also noticed a surge in IL2 concentration in broilers fed with probiotics. IL2 regulation reflects elevated humoral immunity which is observed to be changed in age-dependent manner (Wang et al., 2015; Zhao et al., 2022). Xu et al. (2014) observed up-regulation of IL4 levels in heat-stressed chickens fed with feed additives. Reports of Yu et al. (2022) and Zhen et al. (2018) echo with the outcomes of the current study, which states that a noticeable down-regulation of pro-inflammatory factors (IL6, INF-γ) was observed in broilers fed with *Bacillus* strain-based probiotics than the control. TLR4 acts as the receptor for lipopolysaccharides, which are the primary component of the gram-negative bacteria’s membrane (Kannaki et al., 2010). TLRs recognize particular microbial substances and induce Th1 cytokine production through the NF-κB pathway (Murch, 2001). This study found reduced TLR4 levels in chickens treated with multi-strain probiotics, which aligns with the findings of Yitbarek et al. (2013). Probiotics can elevate TLR’s signaling, regulate mucosal cell-mediated immune responses and enhance epithelial barrier integrity in poultry birds (Gao et al., 2008). HSP70 is a heat shock protein, which is expressed when the host encounters an unfavorable environmental or pathogenic condition (Gu et al., 2012; Zhang et al., 2015). In this study, HSP70 level was elevated in the test groups supplemented with multi-strain probiotics, which corroborates the results of Hu et al. (2021). The down-regulation and up-regulation of HSP70 are the result of host’s response to various biotic and abiotic stress (Siddiqui et al., 2020). Increased HSP70 concentration also shows high heat stress tolerance because of improved cytoprotective effects (Chauhan et al., 2014). HSP70 also plays a crucial role in helping cells recover from stress. It is involved in repairing cellular damage, refolding of impaired proteins, preventing oxidative stress and programmed cell death (Jiang et al., 2020).

Probiotics enhance humoral responses of broilers (Huang et al., 2004). This study shows supplementation of multi-strain probiotics enhanced lymphocyte proliferation in the chickens compared to the control groups. These findings are consistent with the observations of Tollba and Mahmoud (2009), who reported a significant increase in lymphocyte count in probiotics-fed broilers. Muthusamy et al., (2013, 2020) and Paul et al. (2012) demonstrated that dietary supplementation ofβ-glucan in poultry enhanced lymphocyte production, reduced superoxide anion production by blood neutrophils, and exhibited notable immunostimulatory effects. However, a contrary report shows no impact on systemic humoral responses of broilers after probiotics supplementation (Mountzouris et al., 2010).

In this present experiment, it was observed that supplementation of multi-strain probiotics has shown an apparent elevation in the height of villi and depth of the crypts in the small intestines of poultry. The results reflected the conclusions of Ramlucken et al. (2020), Salim et al. (2013), and Sen et al. (2012), who also reported significantly taller villi in chickens after inclusion of multi-strain probiotics in their diet. The present results indicate enhanced gut morphology, which helped in improved overall growth performances and is most likely the major factor for improved FCR in treated chickens. Chang et al. (2020) and Samanya and Yamauchi (2002) also observed improvement in intestinal microflora in broilers administered with multi-strain probiotics. This shows that the enhancement of the villi heights and crypt depths expands the epithelial surface area, promoting more efficient absorption and maximum bioavailability of beneficial nutrients.

Mixed-strain and mixed-species probiotics function in various areas of the gut and perform different action pathway that together generates a combined beneficial effect (Timmerman et al., 2004; Kazemi et al., 2019). Several reports also stated that administration of the mixed-strain probiotics in the diet of poultry, like *Lactobacillus* spp., can elevate the population of favorable microflora and create a healthy gut environment (Khaksefidi and Rahimi, 2005; Swain et al., 2014; Hossain et al., 2015). Reports showed that broilers fed with 2 commercially available multi-strain probiotics displayed improved overall growth performance, enhanced intestinal microflora, and reduced lipid peroxidation (Kazemi et al., 2019). A study investigated that the probiotics mixtures enhanced feed conversion, immune responses, intestinal morphology, and inhibited the pathogenic bacteria such as *E. coli*, *C. jejuni* from colonizing the GI tract (Teo and Tan, 2007).

## Conclusion

The present investigation revealed that inclusion of multi-strain probiotics (*Bifilac^R^*) in poultry diet improved their growth performance, physiological functions, and immunological functions. These findings can help reducing the dependency on AGPs and furthermore probiotics can be suggested as an influential alternative benefiting poultry birds and indirectly humans. Further research is recommended to establish standardized dosages and to identify the beneficial strains of probiotics bacteria, ensuring their optimal production and extensive applicability on a larger scale.

## Data Availability

The raw data supporting the conclusions of this article will be made available by the authors, without undue reservation.
